# Resveratrol-Loaded Lipid Nanocarriers: Correlation between In Vitro Occlusion Factor and In Vivo Skin Hydrating Effect

**DOI:** 10.3390/pharmaceutics9040058

**Published:** 2017-12-10

**Authors:** Lucia Montenegro, Carmela Parenti, Rita Turnaturi, Lorella Pasquinucci

**Affiliations:** 1 Department of Drug Sciences, Pharmaceutical Technology Section, University of Catania, Viale A. Doria 6, 95125 Catania, Italy; 2 Department of Drug Sciences, Pharmacology and Toxicology Section, University of Catania, Viale A. Doria 6, 95125 Catania, Italy; cparenti@unict.it; 3 Department of Drug Sciences, Medicinal Chemistry Section, University of Catania, Viale A. Doria 6, 95125 Catania, Italy; rita.turnaturi@tiscali.it (R.T.); lpasquin@unict.it (L.P.)

**Keywords:** lipid nanocarriers, skin hydration, occlusion factor, nanoemulsions, SLN, NLC, resveratrol

## Abstract

Lipid nanocarriers show occlusive properties that may be related to their ability to improve skin hydration. The aim of this work was to evaluate the relationship between in vitro occlusion factor and in vivo skin hydration for three types of lipid nanocarriers: nanoemulsions (NEs), solid lipid nanoparticles (SLNs) and nanostructured lipid carriers (NLCs). These lipid nanocarriers were loaded with *trans*-resveratrol (RSV) and incorporated in gel vehicles. In vitro occlusion factor was in the order SLNs > NLCs > NEs. Gels containing unloaded or RSV loaded lipid nanocarriers were applied on the back of a hand of 12 healthy volunteers twice a day for one week, recording skin hydration changes using the instrument Soft Plus. An increase of skin hydration was observed for all lipid nanocarriers (SLNs > NLCs > NEs). RSV loading into these nanocarriers did not affect in vitro and in vivo lipid nanocarriers effects. A linear relationship (*r*^2^ = 0.969) was observed between occlusion factor and in vivo increase of skin hydration. Therefore, the results of this study showed the feasibility of using the occlusion factor to predict in vivo skin hydration resulting from topical application of different lipid nanocarriers loading an active ingredient with no inherent hydrating activity.

## 1. Introduction

Nanocarriers are colloidal systems whose particles or droplets sizes range between 1 and 1000 nm. In the 1960s, the first colloidal systems consisting of water, oil, and surfactants were defined as microemulsions (MEs) [1], a term that is misleading as the droplets’ sizes of these systems are in the nanometric range (10–100 nm). The term nanoemulsions (NEs) has been recently used to define systems similar to microemulsions but differing in their preparation methods and stability [2].

About two decades ago, substituting the oil phase of MEs or NEs with a solid lipid, different research groups obtained a new type of colloidal carriers, defined as solid lipid nanoparticles (SLNs) [3,4]. Being the core of SLNs solid, lipid crystallization may occur during their preparation and storage. This phenomenon may induce expulsion of the incorporated drug into the external medium, thus resulting in poor stability of the formulation. Furthermore, SLNs show low drug loading capacity as the ordered structure of the lipid core can incorporate only small amounts of active ingredients. To overcome these drawbacks, lipid nanoparticles with a less ordered lipid matrix, consisting of mixtures of solid and liquid lipids, were developed and defined as nanostructured lipid carriers (NLCs) [5]. Due to the structure of their lipid core, NLCs showed greater loading capacity and better stability than SLNs. SLNs and NLCs main advantages in comparison with other colloidal systems can be summarized as follows: improvement of drug bioavailability and stability, controlled drug release and targeting, safety, and easy and low cost production [6,7,8,9,10]. Therefore, SLNs and NLCs have been proposed for different administration routes (parenteral, topical, and oral).

As far as topical administration is concerned, an additional and interesting feature of SLNs and NLCs is their occlusive effect, which has been attributed to their peculiar properties [11,12,13,14,15]. As reported in the literature [16], occlusive vehicles may improve skin hydration owing to their ability to prevent water loss from the skin layers. An increase of skin hydration could be useful both to treat skin disorders involving water loss and to enhance active ingredient skin permeation [17,18,19]. Wissing et al. [11,12] observed a relationship between in vitro occlusion effects and the following SLNs parameters: size, crystallinity and lipid concentration. In particular, these authors reported an increment of the occlusion factor when SLNs crystallinity or lipid concentration increased or when particle size decreased. SLNs occlusive properties were attribute to their ability to form a continuous and dense film on the skin surface, which prevented water loss from the cutaneous tissue.

However, a recent study, evaluating in vitro occlusive properties and in vivo occlusion effects of SLNs did not find a relationship between particle size and in vivo occlusion effects [20]. As regards NLCs, Loo et al. [13] observed an enhancement of skin hydration increasing NLCs lipid content of nanoparticles showing similar sizes.

To date, the effects of lipid nanocarriers having different matrix composition on in vitro occlusion factor and in vivo skin hydration have not been fully investigated.

The aim of this work was to assess the relationship between in vitro occlusion factor and in vivo skin hydration of three different types of lipid nanocarriers, NEs, SLNs and NLCs, in gel vehicles. All these nanocarriers were prepared using as solid lipid cetyl palmitate, a synthetic wax of generally regarded as safe (GRAS) status, and as liquid lipid isopropyl myristate, a synthetic oil commonly used in pharmaceutical and cosmetic formulations. To evaluate the effect of loading an active ingredient in these nanocarriers, we used *trans*-resveratrol (RSV) as a model drug. RSV (3,5,4′-trihydroxystilbene) is a potent antioxidant produced by many plants as defense against environmental hazards, which has been proposed for pharmaceutical and cosmetic use due to its many biological properties, including anti-inflammatory, cardioprotective, neuroprotective, anticancer, and antiaging activities [21,22,23,24]. RSV has already been loaded into SLNs and NLCs to improve its skin delivery [25], and no inherent hydrating effect has been observed for this active ingredient. Therefore, RSV was supposed not to interfere with lipid nanocarriers effects on skin hydration.

As a linear relationship was observed between in vitro occlusion factor and in vivo skin hydration, the results of this study suggest that determining in vitro occlusion factor could be a useful tool to predict the effect on skin hydration of lipid nanocarriers containing active ingredients with no intrinsic hydrating properties.

## 2. Materials and Methods

### 2.1. Materials

Carbopol Ultrez 21^®^ (Carbopol), *trans*-Resveratrol (RSV), Cetyl Palmitate (CP), Glyceryl Oleate (GO). and Triethanolamine (TEA) were bought from ACEF (Fiorenzuola D’Arda, Italy). Polyoxyethylene-20-oleyl ether (Brij 98^®^, Oleth-20) was bought from Sigma-Aldrich (Milan, Italy). Isopropyl myristate (IPM) was purchased from Farmalabor (Bari, Italy). Methylchloroisothiazolinone and methylisothiazolinone (Kathon CG^®^) and Imidazolidinyl urea (Gram 1^®^) were kindly supplied by Sinerga (Milan, Italy). Distilled water was used throughout this study.

### 2.2. Lipid Nanocarriers Preparation

The composition of unloaded and RSV-loaded NEs, SLNs, and NLCs is reported in Table 1. All nanocarriers were prepared using the phase inversion temperature (PIT) method, as previously reported [26,27]. The aqueous phase contained 0.35% (*w*/*w*) imidazolidinyl urea and 0.05% (*w*/*w*) methylchloroisothiazolinone and methylisothiazolinone as preservatives while the oil phase consisted of lipids, emulsifier, co-emulsifier and different percentages *w*/*w* of RSV. Briefly, the aqueous phase and the oil phase were separately heated at 85 °C and then the water phase was slowly added to the oil phase under stirring. The resulting colloidal system was cooled to room temperature, under constant agitation to obtain lipid nanocarriers.

### 2.3. Lipid Nanocarriers Characterization

#### 2.3.1. Transmission Electron Microscopy

Transmission electron microscopy (TEM) images were acquired by negative-staining electron microscopy. Five µL of colloidal dispersions were put on a 200-mesh formvar copper grid (TAAB Laboratories Equipment, Berks, UK). After sample absorption, the surplus was removed using filter paper, and a drop of 2% (*w*/*v*) aqueous solution of uranyl acetate was added. After surplus removal, the sample was dried at room temperature and then analyzed by a transmission electron microscope (model JEM 2010, Jeol, Peabody, MA, USA) operating at an acceleration voltage of 200 kV.

#### 2.3.2. Size and Zeta Potential Determination

Mean particle sizes and polydispersity indexes (PDI) of NEs, SLNs, and NLCs were determined by dynamic light scattering (DLS) using a Zetasizer NanoZS (ZEN 3600, Malvern, Herrenberg, Germany).

All the samples were diluted 1:5 (sample:double distilled water) and thermostated at 25 °C for 2 min prior to analysis by means of a 4 mW laser diode operating at 670 nm. Zeta (ζ) potential was determined by laser Doppler velocimetry using the same instrument described above. Samples were analyzed according to a procedure already reported, after dilution with KCl 1 mM (pH 7.0) [28].

Each measurement was performed in triplicate and values were expressed as mean ± S.D.

#### 2.3.3. Differential Scanning Calorimetry Analyses

Differential scanning calorimetry (DSC) analyses of NEs, SLNs and NLCs samples were carried out on a Mettler TA STAR^e^ System equipped with a DSC 822^e^ cell and a Mettler STAR^e^ V8.10 software (Mettler-Toledo, Milan, Italy). The instrument calibration (transition temperature and enthalpy changes) was performed using indium and palmitic acid (purity ≥ 99.95% and ≥ 99.5%, respectively; Fluka, Buchs, Switzerland). Then, 100 µL of each sample was hermetically sealed into a calorimetric pan, and DSC analyses were undertaken heating the sample from 5 to 65 °C (rate 2 °C/min) and then cooling from 65 to 5 °C (rate 4 °C/min) at least three times. The reference pan was filled with 100 µL of the same aqueous phase used to prepare the lipid nanocarriers under investigation. The crystallinity index of the lipid nanocarriers was calculated as previously reported [29]. The melting enthalpy of the lipid in the nanocarriers was expressed as percentage of the melting enthalpy of the bulk lipid. Cetyl palmitate melting enthalpy was determined in a previous work and was used as reference (100%) as the bulk lipid was considered being fully crystalline [30]. IPM, being liquid, did not show any melting peak, and its crystallinity index was considered equal to zero. Each experiment was run in triplicate.

#### 2.3.4. Stability Tests

After their preparation, samples of NEs, SLNs, and NLCs were kept in the dark and at room temperature in airtight jars for two months.

At fixed intervals (24 h, one week, two weeks, one month, and two months after their preparation), particle size and polydispersity index of the samples were measured.

### 2.4. Gel Preparation

Gel formulations were prepared by dispersing Carbopol Ultrez 21 (0.8% *w*/*w*) in the selected colloidal dispersions and the mixture was left in the dark at room temperature for 24 h, to allow complete hydration of the gelling agent. Then, the neutralizing agent (TEA, 0.8% *w*/*w*) was added dropwise, and the dispersion was slowly and continuously stirred until the gel was formed. Gels were kept in the dark and at room temperature in airtight jars until use. The following gel formulations were prepared: (1) gel A, without lipid nanocarriers (the aqueous phase consisted of deionized water containing the same preservatives used to prepare lipid nanocarriers); (2) gel SLN, containing unloaded SLNs; (3) gel SLN RSV, containing SLNs loading RSV 1% *w*/*w*; (4) gel NLC, containing unloaded NLCs; (5) gel NLC RSV, containing NLCs loading RSV 1% *w*/*w*; (6) gel NE, containing unloaded NEs; (7) gel NE RSV, containing NEs loading RSV 1% *w*/*w*. Gel A was used as control for occlusion factor determinations and in vivo evaluation of skin hydration. A gel containing only free RSV was not used as control due to the poor water solubility of this compound that prevented us from obtaining a homogenous gel even adding low amounts of solubilizing agents.

### 2.5. Occlusion Factor Determination

The occlusion factor (*F*) was determined according to the procedure reported by Wissing et al. [11]. Beakers (100 mL) were filled with distilled water (50 mL), covered with filter paper (cellulose acetate filter, 90 mm, VWR, Fontenay sous Bois, France, cutoff size 4–7 µm) and carefully sealed with teflon tape. Each gel formulation (200 mg) was spread evenly with a spatula on the filter surface to obtain a thin film formation, which was identifiable throughout the test. The samples were kept at 32 °C (skin temperature) for 48 h (50%–55% relative humidity) in an incubator (Incubator IN 30, Memmert GmbH, Schwabach, Germany). Beakers containing 50 mL of distilled water and covered with filter paper but without an applied sample were used as reference. The samples were weighed after 48 h to determine water loss due to evaporation through the filter.

The occlusion factor (*F*) was calculated as follows:

*F* = 100 × (*A*−*B*)/*A* (1)

where *A* is the water loss without sample (reference) and *B* is the water loss with sample. Each experiment was carried out in triplicate.

### 2.6. In Vivo Evaluation of Skin Hydration

Skin hydrating effects of unloaded and RSV loaded lipid nanocarriers were assessed in vivo from gel vehicles after topical application for one week. Twelve healthy female volunteers (average age 50 ± 3) were selected, and they gave their written informed consent to participate in this study. Younger healthy subjects were excluded from the study due to their highly hydrated skin, which did not allow a proper evaluation of skin hydrating treatments. All participants did not suffer from any dermatological disease and showed normal/moderate dry skin. In vivo evaluations were performed following the rules of the Declaration of Helsinki of 1975, revised in 2008. The local ethics committee declared that no approval was needed for this type of study due to the non-invasive nature of the study and the safety of the ingredients used in the formulations under investigation. The volunteers were instructed how to apply the formulations and were provided with freshly prepared gel samples in nontransparent containers labeled with alphanumeric codes. The participants were asked to apply about 2 mg of gel onto two different areas over the back of their hands, twice a day (morning and evening) for one week. The study was performed in double blind and in two steps. In the first step, the subjects applied four out of the six different gels, and in the second step, the same volunteers tested the remaining two gels, after a one-month washout.

The volunteers did not apply the formulation the morning when the final measurement had to be taken. Skin hydration values were determined prior to the treatment and after one week of treatment. Measurements were taken after 30 min of acclimatization in a room under controlled conditions (temperature 22 ± 1 °C; humidity 35% ± 5%). Only normal hygiene regimen was allowed during the study, and the use of other cosmetic or dermatological products was not permitted. Skin hydration was evaluated using the specific probe of the instrument Soft Plus (Callegari Srl, Parma, Italy), as previously reported [31]. The instrument determined skin hydration by capacity measurements (range 0–100 u.c. (arbitrary units), resolution 1 u.c., precision 5%). Each measurement was performed in triplicate.

Statistical analysis of the results was performed using Students’ *t* test (*p* < 0.05).

## 3. Results and Discussion

### 3.1. Lipid Nanocarriers Characterization

As small-sized lipid nanocarriers are regarded as more occlusive than large-sized ones, in this work we used the PIT method for NEs, NLCs, and SLNs preparation owing to the ability of this method to provide small-sized lipid nanocarriers [26,27].

Table 2 shows that all unloaded and RSV loaded lipid nanocarriers had mean sizes in the range 26–47 nm. Loading RSV into SLNs slightly increased nanoparticles size up to 1% *w*/*w*. Further increase of RSV loading resulted in turbid colloidal suspensions, which led to a precipitate within 48 h from their preparation. Therefore, SLNs loading capacity for RSV was considered 1% *w*/*w* and this percentage of RSV was used to prepare loaded NEs and NLCs. Other authors evaluated SLNs loading capacity for poor water-soluble compounds determining the maximum amount of drug that could be incorporated into the nanocarriers leading to a clear system without no sign of precipitation [32].

To prepare NEs and NLCs, we used IPM, a liquid ester whose ability to enhance both drug skin permeation and skin hydration is well known. Previous studies reported that IPM showed occlusive properties similar to Vaseline, preventing water loss from the cutaneous tissue [33]. However, in our study, IPM was not in free form in the vehicles, but it was incorporated in droplets (NEs) or in lipid matrices (NLCs). During and after application on the skin surface, these nanocarriers could disaggregate to some extent, releasing their components onto and into the stratum corneum. Therefore, IPM could contribute to the occlusive effect of the nanocarriers in which it is incorporated.

Unloaded NLCs mean sizes and polydispersity index (PDI) values depended on solid lipid/liquid lipid ratio. As shown in Table 2, an increment of IPM content from 1 to 5% *w*/*w* resulted in an increase of both mean particle size and PDI values. As reported in the literature [34], the solid lipid/liquid lipid ratio may affect the interactions among NLCs components, resulting in nanoparticles with different curvature radius and different mean sizes.

**Figure 1:**
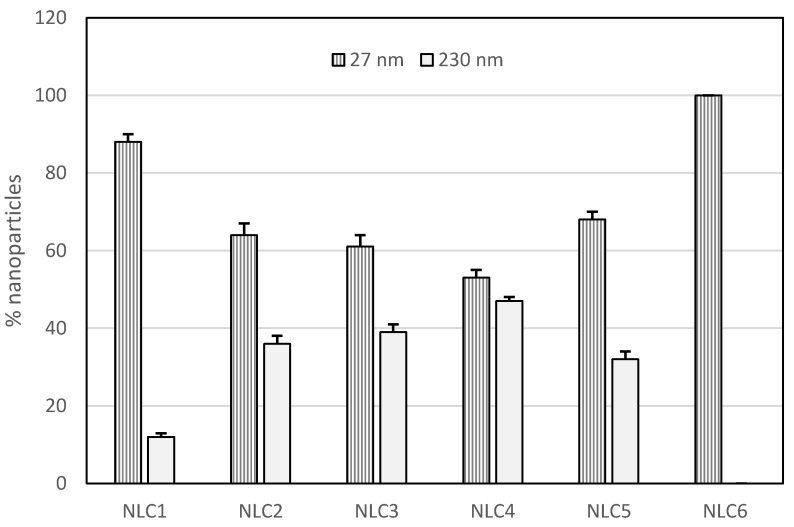
Percentages of nanoparticles with small size (27 nm) and large size (230 nm) in NLC1–6.

As PDI values of NLC1–5 were higher than 0.300, the presence of more than one population of nanoparticles could be expected. Analyzing DLS data, we observed two different populations of lipid nanoparticles in NLC1–5, one with small sizes (mean size 27 nm) and the other with bigger sizes (mean size 230 nm), while NLC6 showed a single population of nanoparticles having small sizes (Figure 1). Samples showing different populations of nanoparticles are not suitable to develop pharmaceutical and cosmetic formulations because they do not show homogeneous physic-chemical and technological properties. Therefore, we did not perform any further investigation on NLC1–5.

Loading RSV 1% *w*/*w* in NLC6 or in NE, no significant change of particles sizes or PDI values was observed in comparison with the corresponding unloaded nanocarriers.

As shown in Table 2, all unloaded and RSV loaded lipid nanoparticles showed similar slightly negative zeta potential values, thus indicating that the incorporation of RSV did not affect their superficial charge.

**Figure 2:**
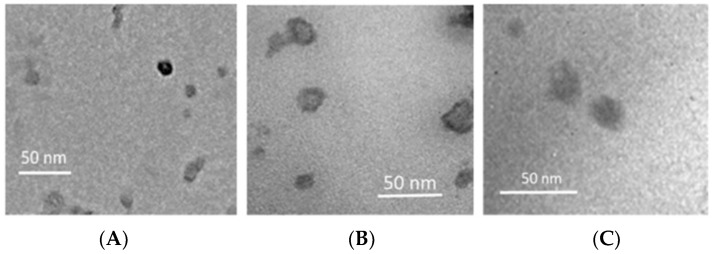
Transmission electron microscopy (TEM) images of lipid nanocarriers containing RSV 1% *w*/*w*. (**A**) NE RSV; (**B**) SLN RSV; (**C**) NLC6 RSV.

Nanocarriers morphology was analyzed by TEM, showing that all nanoparticles were roughly round-shaped. In Figure 2, TEM images of 1% *w*/*w* RSV loaded NEs, SLNs, and NLC6 are reported. Unloaded lipid nanocarriers provided images similar to those of RSV loaded nanocarriers (images not shown). All nanocarriers produced similar TEM images as this technique gave information only about the external surface of the nanocarriers.

**Figure 3:**
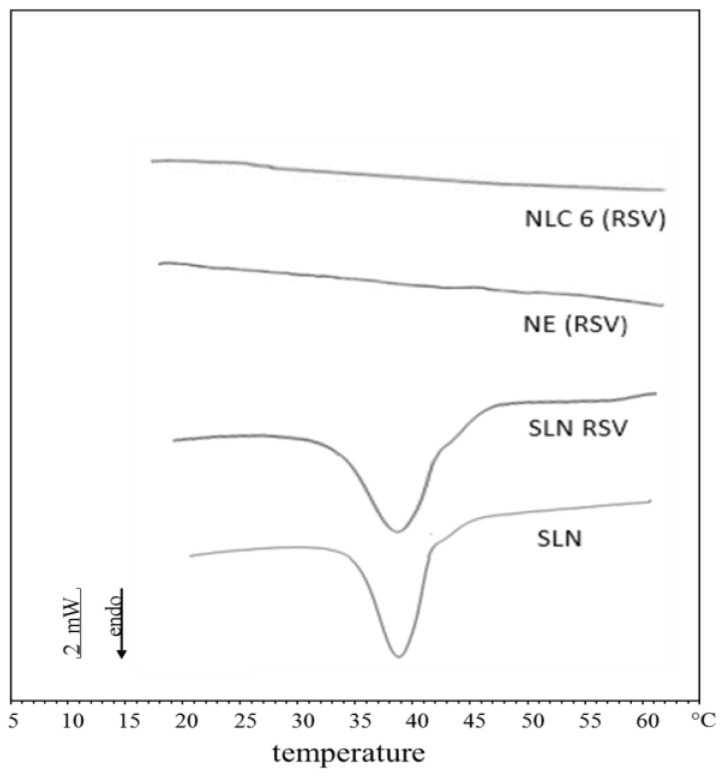
Calorimetric curves, in heating mode, of the following lipid nanocarriers: unloaded SLN (SLN); 1% *w*/*w* RSV loaded SLN (SLN RSV); unloaded NE [NE (RSV)] and 1% *w*/*w* RSV loaded NE [NE (RSV)] curves are overlapping; unloaded NLC6 [NLC6 (RSV)] and 1% *w*/*w* RSV loaded NLC6 [NLC6 (RSV)] curves are overlapping.

To evaluate the crystallinity index of unloaded and 1% *w*/*w* RSV loaded NEs, SLNs and NLC6, we performed DSC analyses on these colloidal suspensions and the thermograms are depicted in Figure 3. As reported in the literature [29], this index is to be regarded as a rough measure due to potentially different forms of nanoparticles crystallization, leading to overlapping peaks whose separation is not possible. For unloaded and RSV loaded NEs and NLC6, no melting peak was observed, thus indicating that the crystallinity of these lipid nanocarriers was equal to zero. On the contrary, both unloaded and RSV loaded SLN showed an evident endothermic peak, corresponding to a crystallinity index of 65% and 59%, respectively. In a previous study, we obtained a similar crystallinity index value for unloaded SLN whose matrix consisted of cetyl palmitate [30]. The lower crystallinity index of RSV SLN suggest that drug loading in these SLN reduced the ordered packing of the lipid matrix of these nanocarriers, as reported by other authors incorporating different active ingredients in SLN [35].

Stability tests, performed at room temperature for two months, did not show any significant change of sizes and PDI values for all investigated nanocarriers (unloaded and 1% *w*/*w* RSV loaded NEs, SLNs, and NLC6, data not shown). To carry out in vitro occlusion tests and in vivo skin hydration evaluations, we incorporated these nanocarriers in a gel vehicle. During their storage (one month at room temperature and in the dark), these gels did not show significant changes of their organoleptic characteristics.

### 3.2. Determination of In Vitro Occlusion Factor

**Figure 4:**
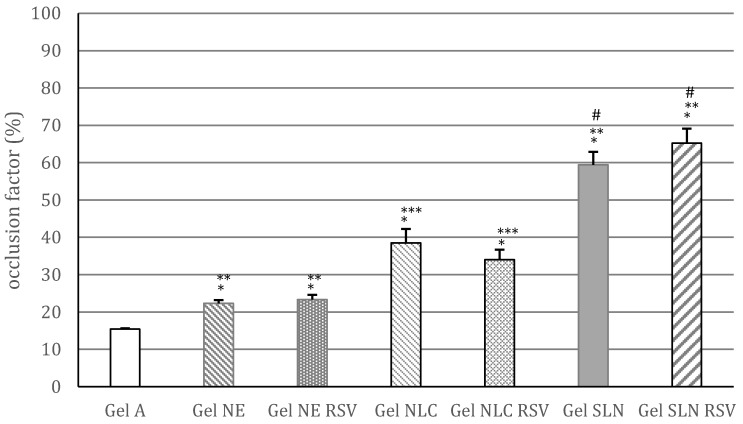
Occlusion factor (%) of gels without lipid nanocarriers (gel A), containing unloaded NE (gel NE), NLC6 (gel NLC), SLN (gel SLN) or 1% *w*/*w* RSV loaded NE (gel NE RSV), NLC6 (gel NLC RSV) and SLN (gel SLN RSV). Statistical analysis for the comparison: * *p* < 0.05 vs. gel A (control); ** *p* < 0.05 vs. all other gels; *** *p* < 0.05 vs. all other gels; # *p* < 0.05 vs. all other gels; *p* > 0.05 for the comparisons: gel NE vs. gel NE RSV, gel NLC vs. gel NLC RSV, gel SLN vs. gel SLN RSV.

As the occlusion factor of SLN and NLC depends on their particles sizes, to evaluate the occlusive properties of lipid nanocarriers with different matrix composition, in this work, we tested lipid nanocarriers with similar sizes (26–36 nm). Figure 4 shows that gel A had a slight occlusive effect, likely due to the presence of the rheological additive, which may form a thin film on the application surface. However, this occlusion factor was significantly lower (*p* < 0.05) than that observed for all the gels containing unloaded or RSV loaded lipid nanocarriers. In particular, the highest occlusion factor was obtained for gels containing SLNs, regardless of the incorporation of RSV. For the gels prepared with non-crystalline nanocarriers (unloaded and RSV loaded NE and NLC6), an evident occlusive effect was obtained, which was significantly higher (*p* < 0.05) for gel NLC and gel NLC RSV in comparison with gel NE and gel NE RSV. Loading RSV into NE or NLC6 did not affect their occlusion factor, similarly to the SLNs. These results indicate that, in addition to the crystallinity of the lipid matrix, the content of solid lipid may affect the occlusive properties of lipid nanocarriers, as previously reported in the literature [11]. However, it is interesting to note that the gels containing non-crystalline nanocarriers without solid lipid (gel NE and gel NE RSV) showed an occlusion factor significantly higher (*p* < 0.05) than the control gel (without lipid nanocarriers), suggesting that the liquid lipid (IPM) may contribute to the occlusive properties of the nanocarriers. Therefore, further studies are planned to evaluate the effects of different types of liquid lipids on the occlusion factor of NEs and NLCs.

### 3.3. In Vivo Evaluation of Gel Formulations

The most commonly used vehicles for topical application of SLNs and NLCs are gels and emulsions [11]. In this work, we chose gel formulations as vehicle for unloaded and RSV loaded lipid nanocarriers to prevent possible interactions between vehicle components and lipid nanocarriers, which may occur in complex vehicles such as emulsions. Furthermore, Souto et al. [36] demonstrated that SLNs and NLCs incorporation in hydrogels did not significantly affect their particles sizes and zeta potential.

**Figure 5:**
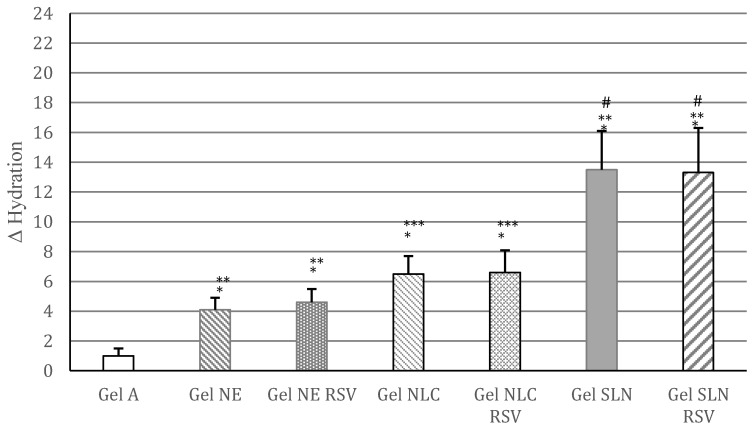
Difference of skin hydration values (Δ hydration) recorded after one-week in vivo topical treatment with the gels under investigation (*n* = 12). Statistical analysis for the comparison: * *p* < 0.05 vs. gel A (control); ** *p* < 0.05 vs. all other gels; *** *p* < 0.05 vs. all other gels; # *p* < 0.05 vs. all other gels; *p* > 0.05 for the comparisons: gel NE vs. gel NE RSV, gel NLC vs. gel NLC RSV, gel SLN vs. gel SLN RSV.

The differences of skin hydration (Δ hydration), recorded after one-week in vivo topical treatment with the gels under investigation, are shown in Figure 5. Gel A, which did not contain lipid nanocarriers, provided only a very slight increase of skin hydration. On the contrary, topical application of gels containing lipid nanocarriers led to a significant increment of skin hydration in the following order SLNs > NLCs > NEs. However, no significant difference (*p* > 0.05) was observed between gels containing unloaded and the corresponding RSV loaded lipid nanocarriers, thus suggesting that RSV incorporation in these nanocarriers was not able to improve further skin hydration. Therefore, although RSV loading into SLN and NLC could be a useful strategy to improve RSV topical antioxidant activity [25,37], this approach did not bring any benefit to skin hydration in a short-term treatment.

Our results confirm the high skin hydrating effect of SLNs previously reported in other works [31,38], pointing out that the solid lipid content is an important feature in the design of lipid nanocarriers intended for improving skin hydration. It is noteworthy that the slight decrease of crystallinity index observed loading RSV into SLNs did not affect skin hydration, thus suggesting that only significant changes of this parameter may provide evident effects on skin hydration. It would have been interesting to evaluate the effect on skin hydration of NLCs prepared with different concentrations of solid lipid. Unfortunately, in our work, these NLCs showed a non-homogenous nanoparticle distribution that make them unsuitable for practical use. Therefore, we planned further studies using different mixtures of liquid and solid lipid to obtain NLCs with suitable technological properties to test the effect of liquid lipid/ solid lipid ratio on skin hydration.

**Figure 6:**
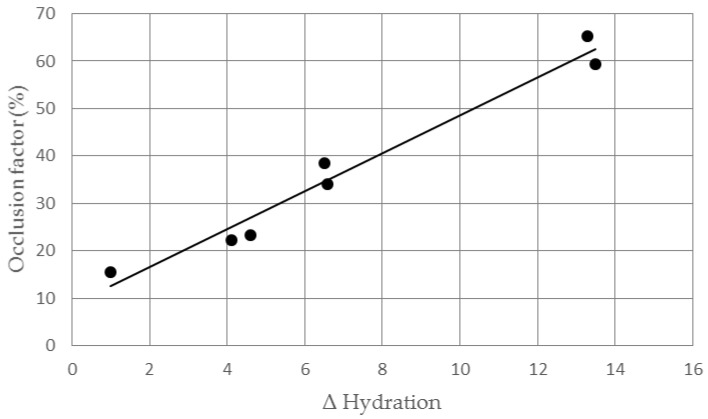
Relationship between in vitro occlusion factor and in vivo Δ hydration for the gels under investigation.

Analyzing in vivo results, we noted a trend similar to that observed in the in vitro occlusion test, as the higher the skin hydrating effect, the higher the occlusion factor. Plotting Δ hydration value *vs* occlusion factor for each gel, we observed a linear relationship (*r*^2^ = 0.969, Figure 6), which suggested that the occlusion factor could be predictive of the skin hydration effects of lipid nanocarriers whose loaded active ingredient had no inherent hydrating activity in a short-term in vivo study.

In conclusion, for lipid nanocarriers with similar sizes, the content of solid lipid seemed to play a key role in determining their occlusive properties and a linear relationship between in vitro occlusion effect and in vivo skin hydration was observed. However, other factors, including the degree of crystallinity of the lipid matrix, the nanocarriers sizes and the type of liquid lipid, could significantly contribute to the occlusive effect. Therefore, the results of this study pointed out that a thorough understanding of the factors affecting the occlusive properties of lipid nanocarriers could be helpful in designing topical formulations with improved effectiveness.

## Figures and Tables

**Table 1:** Composition (% *w*/*w*) of the lipophilic phase of unloaded and resveratrol (RSV) loaded lipid nano-carriers. GO = glyceryl oleate; CP = cetyl palmitate; IPM = isopropyl myristate.

Code	Ingredients				
	Oleth-20	GO	CP	IPM	RSV
SLN	9.0	5.0	7.0	-	-
SLN1 RSV	9.0	5.0	7.0	-	0.5
SLN2 RSV	9.0	5.0	7.0	-	0.7
SLN3 RSV	9.0	5.0	7.0	-	1.0
NLC1	9.0	5.0	6.0	1.0	-
NLC2	9.0	5.0	5.0	2.0	-
NLC3	9.0	5.0	4.0	3.0	-
NLC4	9.0	5.0	3.0	4.0	-
NLC5	9.0	5.0	2.0	5.0	-
NLC6	9.0	5.0	1.0	6.0	-
NLC6 RSV	9.0	5.0	1.0	6.0	1.0
NE	9.0	5.0	-	7.0	-
NE RSV	9.0	5.0	-	7.0	1.0

**Table 2:** Mean sizes (Z-Ave ± S.D.), polydispersity index (PDI ± S.D.) and ζ potential (±S.D.) of unloaded and RSV loaded lipid nano-carriers.

Code	Z-Ave ± S.D. (nm)	PDI ± S.D.	ζ Potential ± S.D. (mV)
SLN	35.76 ± 1.23	0.221 ± 0.003	−1.83 ± 0.31
SLN1 RSV	36.40 ± 1.26	0.202 ± 0.002	−1.99 ± 0.39
SLN2 RSV	47.14 ± 1.39	0.273 ± 0.005	−2.15 ± 0.41
SLN3 RSV	45.99 ± 1.30	0.231 ± 0.001	−2.09 ± 0.40
NLC1	28.92 ± 0.66	0.361 ± 0.003	−1.88 ± 0.35
NLC2	37.43 ± 0.84	0.423 ± 0.002	−2.19 ± 0.41
NLC3	42.73 ± 1.20	0.443 ± 0.019	−1.79 ± 0.55
NLC4	43.77 ± 2.47	0.490 ± 0.034	−2.03 ± 0.62
NLC5	35.80 ± 1.31	0.443 ± 0.041	−2.21 ± 0.46
NLC6	27.24 ± 1.20	0.218 ± 0.006	−2.03 ± 0.51
NLC6 RSV	26.14 ± 1.04	0.215 ± 0.003	−1.98 ± 0.37
NE	30.16 ± 1.17	0.259 ± 0.005	−1.78 ± 0.44
NE RSV	27.08 ± 1.36	0.207 ± 0.006	−1.96 ± 0.31
