# Expression of Concern: Montenegro et al. Resveratrol-Loaded Lipid Nanocarriers: Correlation Between In Vitro Occlusion Factor and In Vivo Skin Hydrating Effect. *Pharmaceutics* 2017, *9*, 58

**DOI:** 10.3390/pharmaceutics18101331

**Published:** 2026-10-20

**Authors:** Pharmaceutics Editorial Office

**Affiliations:** MDPI AG, Grosspeteranlage 5, 4052 Basel, Switzerland; pharmaceutics@mdpi.com

With this notice, the *Pharmaceutics* Editorial Office alerts readers to concerns regarding this article [1].

Following publication, concerns were raised regarding the accuracy of Figure 2. In accordance with the journal’s standard procedures, the Editorial Office and Editorial Board assessed the concerns and sought clarification from the authors. However, despite repeated attempts to obtain additional information, the concerns could not be conclusively resolved, because the Editorial Office was no longer able to establish contact with the authors.

Based on the information available, the Editorial Board concluded that the concerns are limited to Figure 2 and do not call into question the overall findings or conclusions presented in the article.

This Expression of Concern is issued to inform readers of the unresolved concerns regarding Figure 2, which should therefore be interpreted with appropriate caution. No further editorial action is planned at this time. Should additional information become available, however, the Editorial Office and Editorial Board may reconsider the matter and take further action in accordance with MDPI’s policies.

## References

[1] Montenegro L., Parenti C., Turnaturi R., Pasquinucci L. (2017). Resveratrol-Loaded Lipid Nanocarriers: Correlation between In Vitro Occlusion Factor and In Vivo Skin Hydrating Effect. Pharmaceutics.

